# Influence of ewe metabolic status on failure of passive transfer of immunity and lamb production in a UK lowland flock

**DOI:** 10.1002/vetr.5922

**Published:** 2025-11-11

**Authors:** Rob F. Kelly, Amy Jennings, Elizabeth Burrough, Geraldine Russell, Katie Adam, Emily Gascoigne, Peers L. Davies, Jennifer S. Duncan, Andy Hopker, Robert M. Hyde, Fiona M. Lovatt, Ann Bruce, Alexander Corbishley

**Affiliations:** ^1^ Royal (Dick) School of Veterinary Studies and The Roslin Institute University of Edinburgh Roslin UK; ^2^ APHA Field Services Perth UK; ^3^ Synergy Farm Health Dorchester UK; ^4^ Department of Livestock & One Health Institute of Infection Veterinary and Ecological Sciences University of Liverpool Liverpool UK; ^5^ School of Veterinary Medicine and Science University of Nottingham Sutton Bonnington UK; ^6^ School of Social and Political Science University of Edinburgh Edinburgh UK

## Abstract

**Background:**

Inadequate ewe nutrition is an important driver for neonatal lamb losses, although the association of ewe metabolic status with lamb passive transfer status is poorly understood. This study investigates the relationship between ewe metabolic status, neonatal lamb losses, failure of passive transfer of immunity (FPTI) and lamb growth.

**Methods:**

Ewes were blood sampled to assess metabolic status 3 weeks prior to the start of lambing. Within 8‒24 hours of birth, individual lamb weights, sex and litter size were recorded. Lambs were blood sampled to measure serum immunoglobulin G (IgG) to assess FPTI, and lamb outcomes were recorded until weaning.

**Results:**

Ewes had decreased odds of losing a lamb if they were scanned with twins and had a higher plasma albumin concentration. Lambs had an increased risk of FPTI (serum IgG < 24 mg/mL) if they were triplet lambs and if their mothers had increased plasma beta‐hydroxybutyrate concentrations. Lambs with FPTI had increased odds of reduced growth (<0.26 kg/day).

**Limitations:**

This study was conducted in a single, well‐managed, lowland flock. Therefore, the results may not be more widely generalisable.

**Conclusions:**

This study highlights the potential association between ewe nutritional status, FPTI and lamb growth and indicates a need for a consensus IgG cut‐off value for FPTI in lambs.

## INTRODUCTION

Lamb production can be a profitable way to produce meat for human consumption and to sustainably manage natural grassland.^1^, ^2^ Stratification of the UK sheep industry makes the best use of available land to rear sheep, matching sheep breeds to hill, upland or lowland environments.^2^ Lowland flocks are often part of mixed arable or livestock enterprises, with the timing of lambing dependent upon when adequate grazing and supplementary feeding are available. Most lowland production systems utilise the fertile grazing around springtime, enabling higher stocking rates when there is good grass growth to maximise production. Compared to hills and uplands, lowland systems require additional nutritional inputs due to limited pasture availability, such as concentrate feed and pasture fertiliser, to finish lambs efficiently in time for slaughter or sale to store markets.^1^ The profitability of each system is mainly dependent upon minimising lamb losses, particularly during pregnancy or the immediate neonatal period (24 hours postpartum). Where a systemic stressor or an infectious agent is involved, there is often loss of the full litter, with up to 30% of ewes within an individual flock affected.^3^ In pregnancies with multiple embryos or fetuses, loss of an individual conceptus is thought to be common, at approximately 20%.^4^ Lamb mortality during the immediate neonatal period was reported to be between 7% and 10% in the early 2000s.^5^ Although no further studies have investigated lamb mortality in the UK in the past 20 years, anecdotally, similar levels of mortality are commonly reported by farmers to veterinarians in clinical practice. Regardless of the causes of lamb loss, veterinarians are well placed to advise lowland sheep farmers on how to minimise losses through evidence‐based preventive health planning. However, there is a need for better evidence to identify which ewes are at greatest risk of losing a lamb in order to implement targeted health interventions.

Drivers for losses during pregnancy and the immediate neonatal period are multifactorial, including neonatal husbandry, maternal genetics, nutrition and mothering ability.^3^, ^6^ Although the importance of specific risk factors is likely to vary between individual flocks, ewe nutrition is an important determinant of lamb losses during pregnancy and the immediate neonatal period. Twin‐ or triple‐bearing ewes are particularly at risk of losing one or more lambs, as they have a greater demand for protein and energy compared to ewes bearing single lambs.^7^ Partial losses (i.e., loss of a proportion of the embryos from a multiparous pregnancy) in the last trimester are thought to be associated with ewes in poor body condition, although there is limited evidence to identify specific nutritional risk factors.^8^ Furthermore, lambs rely on an adequate supply of colostrum from their dams to provide sufficient nutrition and immunological protection from infections, especially in the first few hours and days of life. Lambs are born hypogammaglobulinaemic, with limited energy reserves,^9^ and must ingest sufficient colostrum for immunological protection in early life. Failure of passive transfer of immunity (FPTI) occurs when lambs receive inadequate transfer of colostral antibodies, and is associated with insufficient quality and/or quantity of colostrum in the first few hours of life.^10^ Lambs that suffer from FPTI are assumed to be at greater risk of morbidity, mortality and reduced growth rates during the neonatal period and beyond.^5^, ^11^ Oral antibiotic administration to lambs at birth has been common practice in intensive lowland flock systems to minimise neonatal losses associated with bacterial infections. Although this practice is useful where there is a high risk of disease,^12^ it is likely overused in some well‐managed UK sheep systems, where neonatal disease prevention through adequate hygiene and colostrum provision may be sufficient.

As maternal nutrition is a potential driver for fetal losses and FPTI, improving our understanding of the metabolic status of ewes prior to lambing and subsequent lamb outcomes may identify ewes likely at risk of lamb production losses.

Using a longitudinal study dataset from a single UK lowland flock, we investigated factors associated with lamb losses (from the last trimester until 24 hours post‐lambing) and FPTI (8‒24 hours of age). Subsequently, we investigated lamb outcomes from turnout at 24‒48 hours of age until weaning.

## MATERIALS AND METHODS

### Study design

A longitudinal observational study was conducted using a lowland sheep flock in Midlothian, UK. The ∼250‐head flock is a composite‐bred flock of Beltex, North Country Cheviot and Texel cross ewes. In early January 2019, pregnant ewes were scanned. Single‐carrying ewes were kept outdoors and offered supplementary haylage ad libitum until lambing. Twin‐ and triplet‐carrying ewes were housed and fed ad libitum haylage (42.2% dry matter [DM], 11.1 MJ/kg DM, 13% crude protein), with a flat rate of concentrate feeding in the last 6 weeks of pregnancy (680 g/head/day of a 12.5 MJ/kg DM, 18% crude protein pellet), until lambing. From 21 March to 29 April 2019, all ewes lambed indoors and were turned out onto pasture 24‒48 hours post‐lambing, after castration and tail‐docking of lambs using a rubber ring. Lambs were not fed creep, with the stock workers rotating nursing ewes and growing lambs to provide sufficient pasture to graze. Ewes nursed lambs for 3‒4 months until weaning. Lambs were then moved onto silage aftermaths and sold in batches from early August.

A sample size calculation was conducted using an online calculator,^13^ based on a study design of two independent study groups with a dichotomous endpoint (alive/dead). Due to limited literature on passive transfer in lambs in the UK, we assumed similarity to cattle and considered insufficient passive transfer to be when the serum immunoglobulin G (IgG) concentration was 24 mg/mL or less, with an approximately twofold association with mortality, as in UK suckler herds.^14^ Assuming a UK average lamb mortality of 10%,^5^ and extrapolating from results in UK suckler cattle, we estimated the expected mortality in lambs with sufficient passive transfer (10%) and FPTI (20%). With a study power of 0.8, an *α* of 0.05 and a *β* of 0.2, the sample size of 429 lambs was calculated. In January 2019, a total of 470 fetuses were scanned in pregnant ewes, suggesting a sufficient number of lambs.

### Data collection

Three weeks prior to lambing, 10 mL of blood was collected into lithium‐heparin vacutainers from all ewes via jugular venepuncture. Ewe data, including age, body condition score (BCS) and number of lambs scanned, were also collected. Body condition scoring was conducted using a five‐point scale, where 1 was thin and 5 was fat.^15^ During lambing, all lambs between 8 and 24 hours of age were double ear tagged and weighed, and 2 mL whole blood samples were taken from the jugular vein for testing to determine their passive transfer status. Lamb sex and birth litter size were also recorded. Lambs were also immediately assigned to either an oral spectinomycin antibiotic (1 mL of 50 mg: Spectam‐scour‐halt) or a placebo (1 mL tap water with yellow food colouring added) treatment group. The oral antibiotic chosen was based on the industry standard for prophylactic antibiotic treatment of lambs for *Escherichia coli* enterotoxaemia (watery mouth), which was known to be present on the study farm during the lambing season. Stock workers were blinded to the treatment type, and lambs were assigned randomly, with a coin tossed to determine which treatment the first lamb in the litter received. When conducting the analysis, researchers were also blinded to the treatment type. When there was a second lamb in the litter, it automatically received the other treatment. If lambs appeared weak or hungry within the first 24 hours postpartum, additional colostrum was given by farm staff (frozen bovine colostrum from a local dairy farm). Ewes only reared lambs they gave birth to, with no lambs ‘fostered’ onto another ewe throughout the study. For triplet litters, the third lamb was removed, reared separately and consequently not included in the study.

Ewes and lambs were turned out when the lambs were 24‒48 hours old, with commercial husbandry practices continuing as usual throughout the study. Records of lamb and ewe deaths or disease incidents that required treatment were kept by farm staff. Records included the animal ear tag identifier, the nature of the disease incident and the treatment given. In accordance with the veterinary health plan, all twin‐rearing ewes were given moxidectin (Cydectin, Zoetis) at turnout. From mid‐May, all lambs received monthly drenches of anthelmintic, including one treatment with levamisole (Chanverm, Chanelle Pharma) and two treatments with albendazole with added cobalt and selenium (Rycoben, Elanco). All lambs were weighed at weaning and moved to silage aftermaths.

### Laboratory methods

Ewe blood samples were processed by the Dairy Herd Health and Productivity Service (DHHPS) at the Royal (Dick) School of Veterinary Studies, University of Edinburgh (R(D)SVS) to assess metabolic status. Individual blood samples were processed on the day of collection and centrifuged at 1008 × g for 10 minutes, with plasma decanted for further testing. The individual metabolic parameters assayed were plasma beta‐hydroxybutyrate (BOHB, as an indicator of short‐term energy balance), urea nitrogen (urea‐N, as a proxy for effective rumen degradable protein supply), albumin (as a measure of long‐term protein balance) and total protein to calculate globulin (as an indicator of inflammation). Samples were analysed using an Instrumentation Laboratory IL600 wet chemistry system, and quality assurance was undertaken using internal and external quality controls.^16^

Lamb blood samples were processed to assess passive transfer status by measuring serum IgG. Once collected, individual blood samples were immediately stored at 2‒4°C. Within 24‒48 hours, samples were centrifuged at 1008 × g for 10 minutes, with serum decanted and stored at ‒20°C until testing. Serum IgG was assessed by the Biomarkers Laboratory at Scotland's Rural College using the ovine IgG sandwich ELISA.^17^ Serum samples were diluted to 1:400,000 with assay diluent. For positive controls, serial dilutions of an ovine IgG standard were conducted to produce known samples of 240, 120, 60, 30, 15, 7.5 and 3.75 ng/mL. Distilled water was used as a negative control. Controls and samples were tested in duplicate on each plate. The means were taken from each duplicate sample and control. A plate passed quality control if the mean negative control optical density (OD) was less than 0.1 and the mean OD for the 240 ng/mL standard was greater than 1.2. For each plate, a standard curve of the known mean control IgG concentrations was plotted against the mean control OD. Standard curves from each plate were used to interpolate sample IgG concentrations from the mean sample ODs.

### Statistical analysis

All records were initially recorded on paper and transferred to spreadsheets (Microsoft Excel). Data merging, cleaning, analysis and plotting were performed in R Studio 0.98^18^ using the *tidyverse* package.^19^ The numerical variables for ewes included metabolic profile parameters, and for lambs, the numerical values included birth weight and initial serum IgG. For ewes, total litter birth weight was calculated as the sum of the weight of all the lambs born in their litter. The numerical variables were first assessed for normality. Normally distributed data were then summarised by calculating the mean and standard deviation (SD), while non‐normally distributed data were summarised by calculating the median and interquartile range (IQR). For the metabolic profile parameters, standard clinical cut‐off values were used to describe the results (as defined by DHHPS; BOHB: high >0.9 mmol/L; urea‐N: low <1.7 mmol/L; albumin: low <30 g/L; globulin: high >50 g/L). All remaining categorical variables were summarised using proportions. As the serum IgG concentration associated with FPTI is undefined in lambs, calf cut‐off values were used to convert serum IgG to a categorical variable to describe FPTI frequency. A recent consensus paper in dairy calves has defined less than 10 mg/mL as poor passive transfer (often termed ‘complete’ FPTI) and greater than 25 mg/mL as excellent passive transfer (with values between 10 and 25 mg/mL often referred to as ‘partial’ FPTI).^20^ In the absence of appropriate ovine cut‐off values, we used these published bovine values. Lambs that survived until weaning had an individual average daily liveweight gain (DLWG), which was calculated as follows:

$$\mathrm{DLWG}=\frac{\mathrm{Weaning}\;\mathrm{weight}\;(\mathrm{kg})-\;\mathrm{birth}\;\mathrm{weight}\;(\mathrm{kg})}{\mathrm{Age}\;(\mathrm{days})}$$

In keeping with key performance indicators proposed by some UK veterinary practitioners,^21^ lamb DLWG was divided into top, middle and bottom thirds, with means and SDs calculated for each group. Scatter plots were used to explore correlations with continuous numerical variables, and, where appropriate, these correlations were analysed further using a linear model.

Multivariable logistic regression (MLR) models were built to investigate factors associated with the following:

1. Losing a lamb between scanning and 24 hours postpartum, based on the ewe dataset with the dichotomous outcome variable ‘losing one or more lamb’. Explanatory variables included ewe age, BCS, number of lambs scanned, total litter birth weight and metabolic profile parameters (BOHB, urea‐N, albumin and globulin).
2. FPTI, as defined by lamb serum IgG 8‒24 hours postpartum. Using the lamb dataset, two models were developed based on published consensus dairy calf serum IgG cut‐off values for FPTI. The two dichotomous outcome variables assessed were complete FPTI (serum IgG 10 mg/mL or less) and partial FPTI (serum IgG 24 mg/mL or less). Explanatory variables included lamb sample date, weight, sex, litter size, ewe age, BCS, total litter birth weight and metabolic profile parameters (BOHB, urea‐N, albumin and globulin).

MLR models were also developed to investigate the dichotomous outcomes of lamb mortality (defined as death during the study), lamb morbidity (defined as a recorded disease incident during the study) and reduced DLWG (defined as less than the mean DLWG of the bottom third of lambs from turnout at 24–48 hours of age until weaning). Lamb DLWG was also investigated as a continuous outcome using linear regression.

For each model, univariable analysis was conducted to explore associations between outcomes and ewe or lamb explanatory variables.^22^ Explanatory numerical (including ewe metabolic values and total litter birth weight; lamb serum IgG, birth/weaning weights and DLWG) and categorical variables were tested using the student's *t*‐test and chi‐squared tests, respectively. The impact of confounding explanatory variables was minimised through randomisation of the sampling process and by restriction through subsetting the dataset to animals of interest, for example, twin lambs.^23^ To assess for interaction between significant explanatory variables, potential biological collinearity was assessed by calculating the phi coefficient using the psych package.^24^ If phi was 0.5 or more, the two variables were considered collinear and the explanatory variable with the highest *p*‐value was selected. Explanatory variables were included in the final MLR model selection if their univariable analysis *p*‐value was 0.2^22^ or less.

All MLR models were developed using the R packages *stats* ^25^ and *lmer* ^21^ using *base* and *glm* functions. To account for lamb clustering by litter, ewe identifier was included as a random effect in all MLR models investigating FPTI. MLR model selection was based on a conservative approach to find the best‐fitting model to describe the dataset. To select the most parsimonious MLR model to fit the data, backwards stepwise selection using the Akaike information criterion (AIC)^26^ was undertaken. Each variable from the MLR model was removed singularly to assess changes in AIC until the lowest AIC was achieved. The *p*‐value and odds ratio (OR) with 95% confidence interval (CI) were also estimated for explanatory variables. Explanatory variables were deemed statistically significant if their OR CI did not include 1 and if the *p*‐value was 0.05 or less. For metabolic parameters, ORs are relative for every 1 unit increase in these variables. Finally, the final model fit was assessed by plotting residuals for validation.

## RESULTS

### Sample description

#### Ewes

**FIGURE 1:**
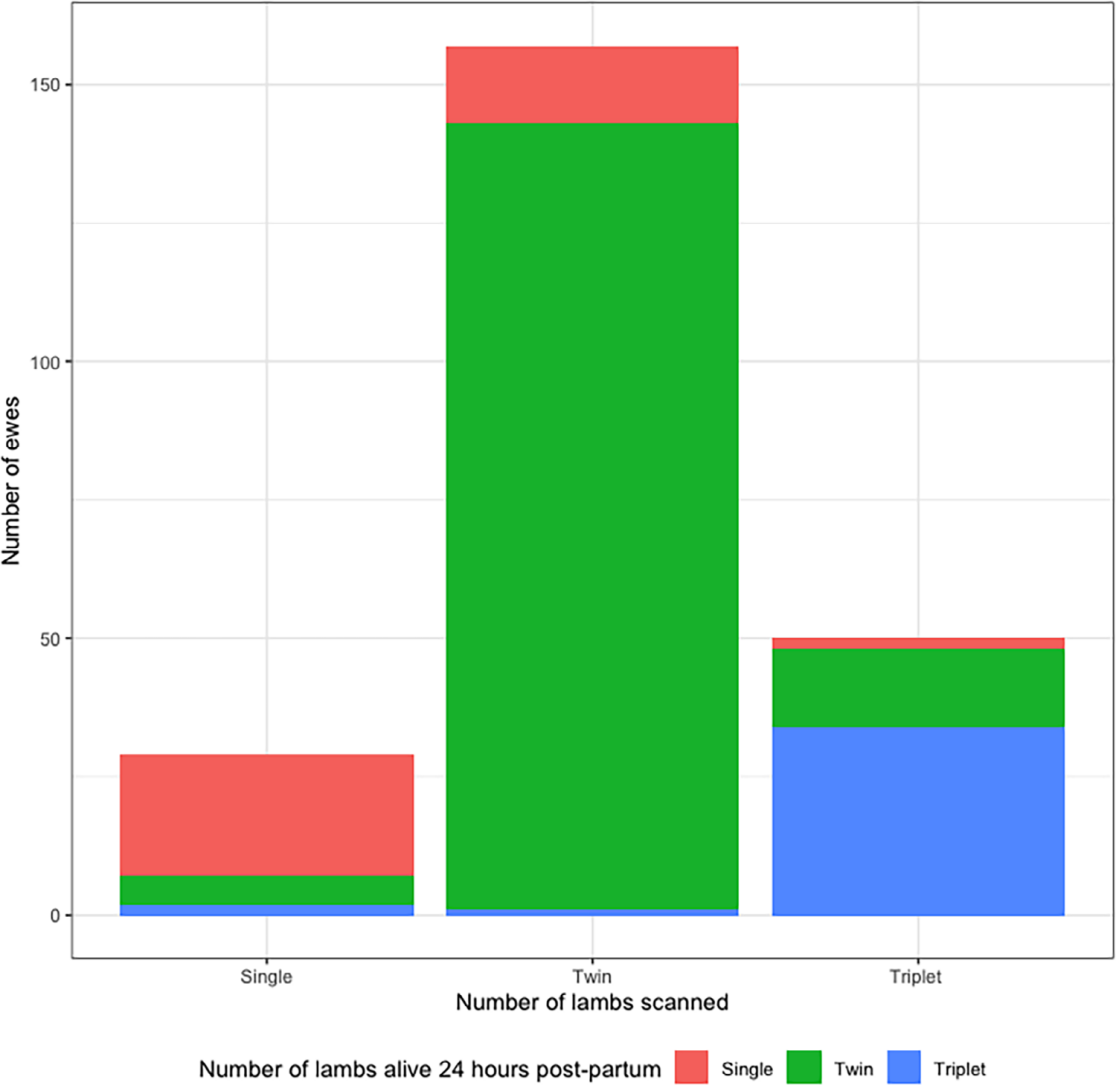
Number of lambs at scanning compared to 8‒24 hours old (*n* = 236). The number of lambs at scanning is given on the *x*‐axis, and the colour of the bar sections represents the number of lambs surviving at 24 hours of age. Note the small number of scanning errors in ewes delivering one or two lambs more than their recorded litter size at scanning.

In total, 252 ewes were enrolled, with 236 ewes included in the analysis. Sixteen ewes were excluded, either because they did not produce any lambs (*n* = 5), their lambs were not weighed within 24 hours of birth (*n* = 9) or they reared triplets throughout the study period (*n* = 2). The ewe cohort sampled is described in Table 1. Of the 471 lambs born to the 236 ewes included in the analysis, 38 were singles, 322 were twins and 111 were triplets. Between scanning and 24 hours postpartum, 0.0%, 9.5% and 37.0% of single‐, twin‐ and triplet‐bearing ewes had a conceptus die, respectively (Figure 1).

**TABLE 1:** Ewe descriptive data (*n* = 236).

Ewe parameter	Percentage (*n*)
Age at lambing (months)	
24	36.9% (87)
36	24.6% (58)
48	22.5% (53)
>48	16.1% (38)
Body condition score	
2	13.1% (31)
3	47.0% (111)
4	39.8% (94)
Number of lambs scanned	
Single	12.3% (29)
Twin	66.5% (157)
Triplet	21.2% (50)
Number of lambs at 24 hours post‐lambing	
Single	16.1% (38)
Twin	68.2% (161)
Triplet	15.7% (37)

**FIGURE 2:**
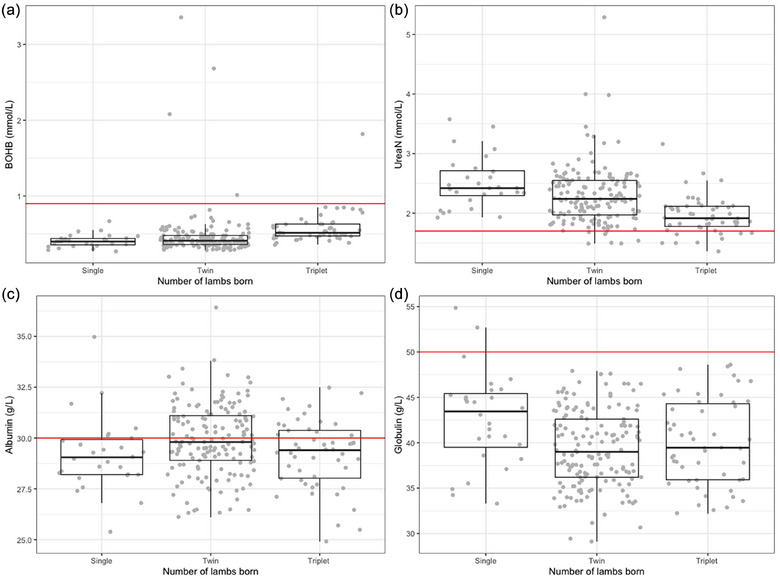
Ewe cohort plasma metabolic profile (*n* = 231). Baseline values, based on optimum values used by the dairy herd health and productivity service to assess group trends, are highlighted by the red horizontal line. These baseline values were not used to define cut‐offs in the analysis of these data; however, they serve as a useful guide for what may be considered a ‘desirable’ value in ewes in late pregnancy. (a) Beta‐hydroxybutyrate (BOHB), (b) urea nitrogen (urea‐N), (c) albumin and (d) globulin.

Ewe metabolic profile parameters are presented in Figure 2, aggregated by litter size (*n* = 231, as five ewes did not have a metabolic profile completed). Interpreting group‐level trends using optimal values, few ewes had elevated BOHB and low urea‐N results, highlighting adequate short‐term energy and protein status in all groups. Globulin results were generally low, indicating possible low levels of inflammatory disease in the whole cohort. Albumin concentration was distributed above and below the cut‐off, particularly for twin‐ and triplet‐carrying ewes. Triplet‐bearing ewes had significantly lower albumin levels than did twin‐bearing ewes (*p* < 0.05).

#### Lambs from birth until 24 hours old

**FIGURE 3:**
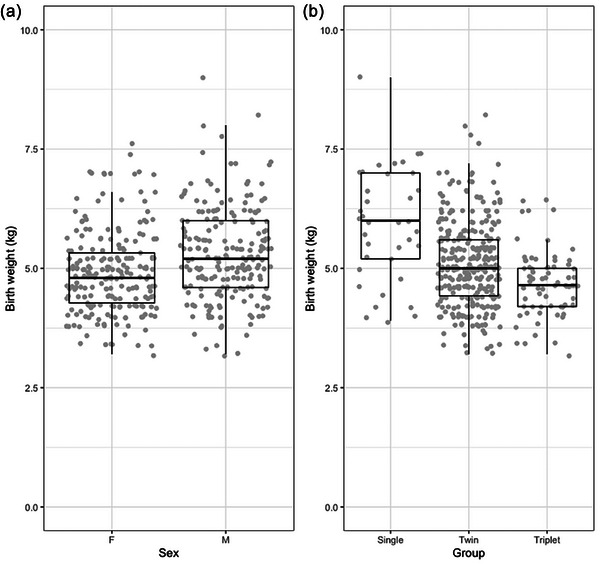
Lamb birth weight (*n* = 413) (a) by sex and (b) by litter size at 24 hours of age.

In total, 413 lambs born from 231 ewes were included in the analysis, and these are described in Table 2. Even numbers of male and female lambs were born, with most being from twin litters. At birth, male lambs were heavier than females (*p* < 0.01). Singles were born heavier than those born as twins and triplets (*p* < 0.01), and those born as twins were heavier than those born as triplets (*p* < 0.01) (Figure 3).

**TABLE 2:** Lamb descriptive data (a) from birth until 24 hours and (b) from 24 hours until weaning (*n* = 413; except for weaning weight).

(a) At birth until 24 hours	
Lamb parameter	Percentage (total)
Sex	
Male	48.7% (201)
Female	51.3% (212)
Sibling (born as)	
Single	9.0% (37)
Twin	74.1% (306)
Triplet	16.9% (70)

	Mean (SD, range)
Birth weight	
Male	5.3 kg (1.0 kg, 3.2–9.0 kg)
Female	4.9 kg (0.9 kg, 3.2–7.6 kg)
Single	5.9 kg (1.2 kg, 3.9–9.0 kg)
Twin	5.1 kg (0.9 kg, 3.2–8.2 kg)
Triplet	4.7 kg (0.7 kg, 3.2–6.4 kg)

(b) From 24 hours until weaning	
Lamb parameter	Percentage (total)
Reared litter size	
Single	9.0% (37)
Twin	91.0% (376)
Antibiotic treatment	
Oral antibiotic	49.9% (206)
Placebo	47.9% (198)
Unknown	2.2% (9)
Additional colostrum	
Yes	15.7% (65)
No	84.3% (348)

	Mean (SD, range)
Birth weight*	
Single	5.9 kg (1.2 kg, 3.9–9 kg)
Twin	5.0 kg (0.9 kg, 3.2–8.2 kg)
Weaning weight*	
Male	38.4 kg (5.8 kg, 19.5–53.0 kg)
Female	37.3 kg (4.9 kg, 25.5–51.5 kg)
Single	42.1 kg (5.0 kg, 28.5–51.5 kg)
Twin	37.4 kg (5.3 kg, 19.5–53.0 kg)

Abbreviation: SD, standard deviation.

* For litter size as reared rather than at birth.

**FIGURE 4:**
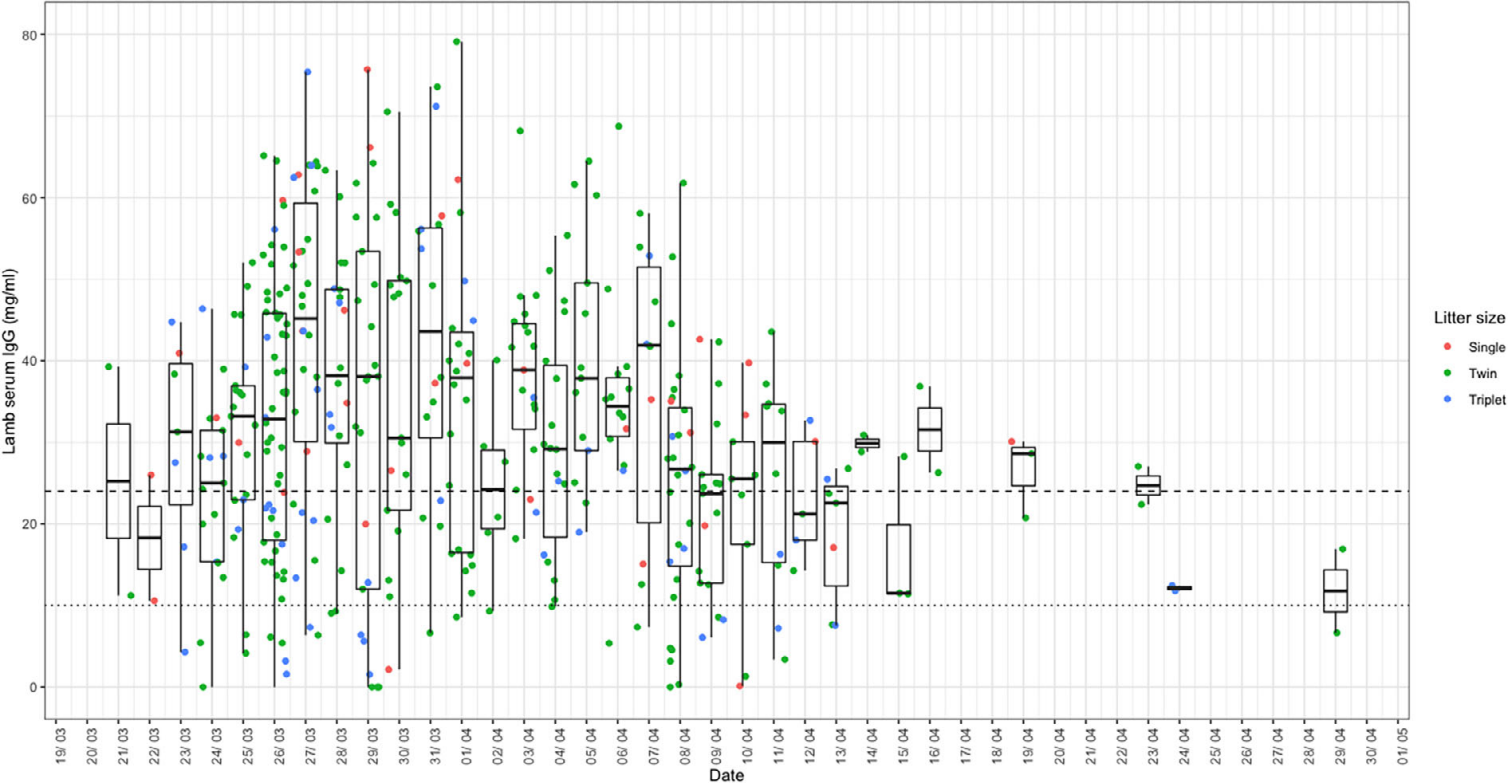
Lamb serum immunoglobulin G (IgG) distribution throughout the lambing period (*n* = 413). The colour of the points represents the litter size recorded at 24 hours of age. Horizontal lines represent cattle cut‐off values for failure of passive transfer of immunity (FPTI): dotted line = complete FPTI, serum IgG 10 mg/mL or less; dashed line = partial FPTI, serum IgG 24 mg/mL or less.

Serum IgG was measured in lambs between 8 and 24 hours postpartum (median = 31.66 mg/mL, IQR 19.71‒44.53 mg/mL). Variation in serum IgG was noted in individual lambs throughout the lambing period (Figure 4). Using the published IgG cut‐off values for defining ‘complete’ (≤10 mg/mL) and ‘partial’ (≤24 mg/mL) FPTI in cattle, 10.4% (43/413) and 33.2% (137/413) of lambs were suspected to have ‘complete’ and ‘complete or partial’ FPTI, respectively.

#### Lambs from 24 hours until weaning

Of the 413 lambs followed from birth through weaning, 4.1% died and 2.6% experienced an episode of clinical disease that required treatment. Of the total reported disease incidents (*n* = 11), watery mouth (45.0%), pneumonia (45.0%) and joint ill (9.0%) were recorded specifically.

**FIGURE 5:**
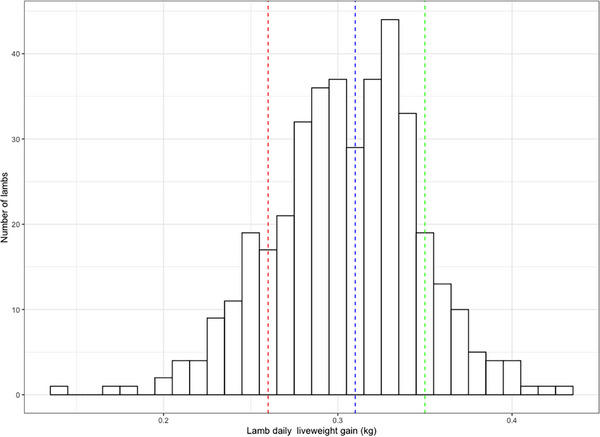
Lamb daily liveweight gain (DLWG) from birth until weaning. The red line indicates the mean of the bottom third of lambs, the blue line indicates the mean of the middle third and green line indicates the mean of the top third when lambs are categorised by DLWG (*n* = 396).

A total of 396 lambs were recorded at weaning, with this subset used for analysis of lamb outcomes. The mean DLWG of these lambs was 0.30 kg/day overall. The cohort was ranked and then split into thirds by DLWG (Figure 5). The mean DLWG of the top third was 0.35 kg/day (range 0.33–0.43 kg/day), the middle third was 0.31 kg/day (range 0.29–0.33 kg/day) and the bottom third was 0.26 kg/day (range 0.14–0.29 kg/day), giving a 0.09 kg/day difference in DLWG between the top and bottom thirds.

### Factors associated with losing a lamb between scanning and 24 hours postpartum

Of ewes with complete metabolic profile results (*n* = 231), 0% (0/28) of single‐bearing, 9.2% (14/153) of twin‐bearing and 32.0% (16/50) of triplet‐bearing ewes lost a lamb between scanning and 24 hours postpartum. As no losses were reported in ewes with single lambs, only ewes scanned with twins or triplets were included for further analysis (*n* = 203).

Univariable analysis investigated relationships between losing a lamb (dichotomous outcome variable: losing one or more lamb) and explanatory ewe variables. Following univariable analysis, the number of lambs scanned (*p* < 0.01), total litter birth weight (*p* < 0.01), ewe albumin (*p* < 0.01) and ewe globulin (*p* < 0.01) were included in the initial MLR model. Total litter birth weight and ewe globulin were removed from the model due to their biological relationship with the number of lambs scanned and albumin values, respectively (phi coefficient ≥0.5). In the final model, ewes had decreased odds of losing at least one lamb if they were scanned with twins (OR = 0.25, 0.00‒0.12, *p* < 0.01) and had a higher plasma albumin concentration (OR = 0.76, 0.00‒0.60, *p* < 0.05; Table 3).

**TABLE 3:** Results of the final multivariable logistic regression model to identify factors associated with ewes losing at least one lamb between scanning and 24 hours postpartum (*n* = 203).

Variable	Level	OR (95% CI)	*p*‐value
Number of lambs at scan	Triplet	Base	
	Twin	0.25 (0.00‒0.12)	<0.01
Ewe plasma albumin (g/L)	As numeric	0.76 (0.00‒0.60)	<0.05

Abbreviations: CI, confidence interval; OR, odds ratio.

### Factors associated with lamb serum IgG 8‒24 hours postpartum

The associations between FPTI (defined by lamb serum IgG at 8‒24 hours of age) and explanatory ewe or lamb factors were initially explored using univariable analysis. None of the variables demonstrated an association with serum IgG as a continuous variable. Therefore, two models were developed based on published dairy calf serum IgG cut‐off values to define FPTI (dichotomous outcome variables were [a] complete FPTI: IgG ≤ 10 mg/mL and [b] partial FPTI: IgG ≤ 24 mg/mL).

For complete FPTI, litter size (*p* < 0.05) and ewe age (*p* < 0.1) were identified as significant in the univariable analysis. Only litter size remained in the final MLR model, but it was not significantly associated with complete FTPI (Table 4). For partial FPTI, litter size (*p* < 0.01) and ewe BOHB (*p* < 0.01) were identified as statistically significant in the univariable analysis. In the final MLR model, triplet lambs (OR = 2.98, 1.01‒8.81, *p* < 0.05) and lambs from ewes with increased BOHB (OR = 5.16, 1.45‒18.39, *p* < 0.05) had a higher odds of partial FPTI (Table 5).

**TABLE 4:** Results of the final multivariable logistic regression model to identify factors associated with lamb serum immunoglobulin G (IgG) concentrations of 10 mg/mL or less (*n* = 413).

Variable	Level	OR (95% CI)	*p*‐value
Litter size at 8‒24 hours post‐lambing	Single	Base	
	Twins	1.29 (0.08‒37.91)	0.51
	Triplets	3.52 (0.04‒142.92)	0.88

Abbreviations: CI, confidence interval; OR, odds ratio.

**TABLE 5:** Results of the final multivariable logistic regression model to identify factors associated with lamb serum immunoglobulin G (IgG) concentrations of 24 mg/mL or less (*n* = 413).

Variable	Level	OR (95% CI)	*p*‐value
Litter size at 8‒24 hours post‐lambing	Single	Base	
	Twins	1.31 (0.52‒3.28)	0.56
	Triplets	2.98 (1.01‒8.81)	<0.05
Ewe plasma BOHB (mmol/L)	As numeric	5.16 (1.44‒18.39)	<0.05

Abbreviations: BOHB, beta‐hydroxybutyrate; CI, confidence interval; OR, odds ratio.

### Factors associated with lamb morbidity and mortality until weaning

Due to the small number of lambs that died or suffered from an episode of disease, we could not use univariable or multivariable analyses to investigate associated risk factors.

### Factors associated with lamb growth until weaning

**FIGURE 6:**
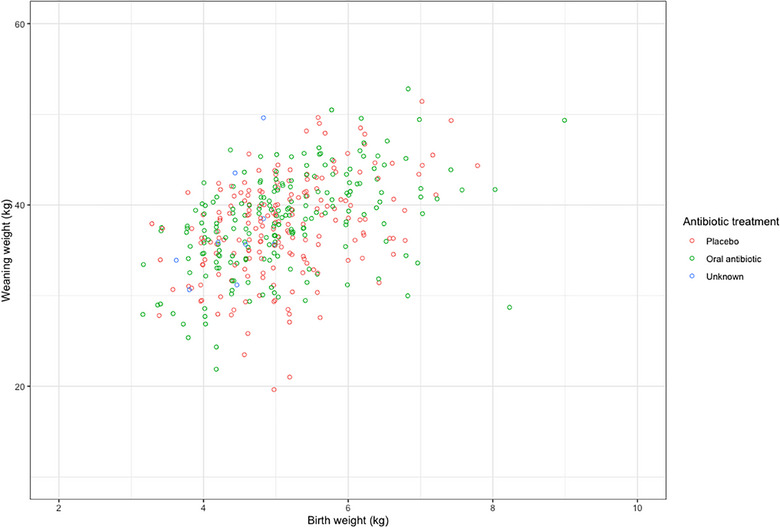
Relationship between lamb birth weight and weaning weight, categorised according to oral antibiotic treatment (*n* = 396). Note the weak relationship between lamb birth weight and weaning weight (*r* ^2^ = 0.19), with no associated relationship with antibiotic treatment (*p* > 0.05).

**FIGURE 7:**
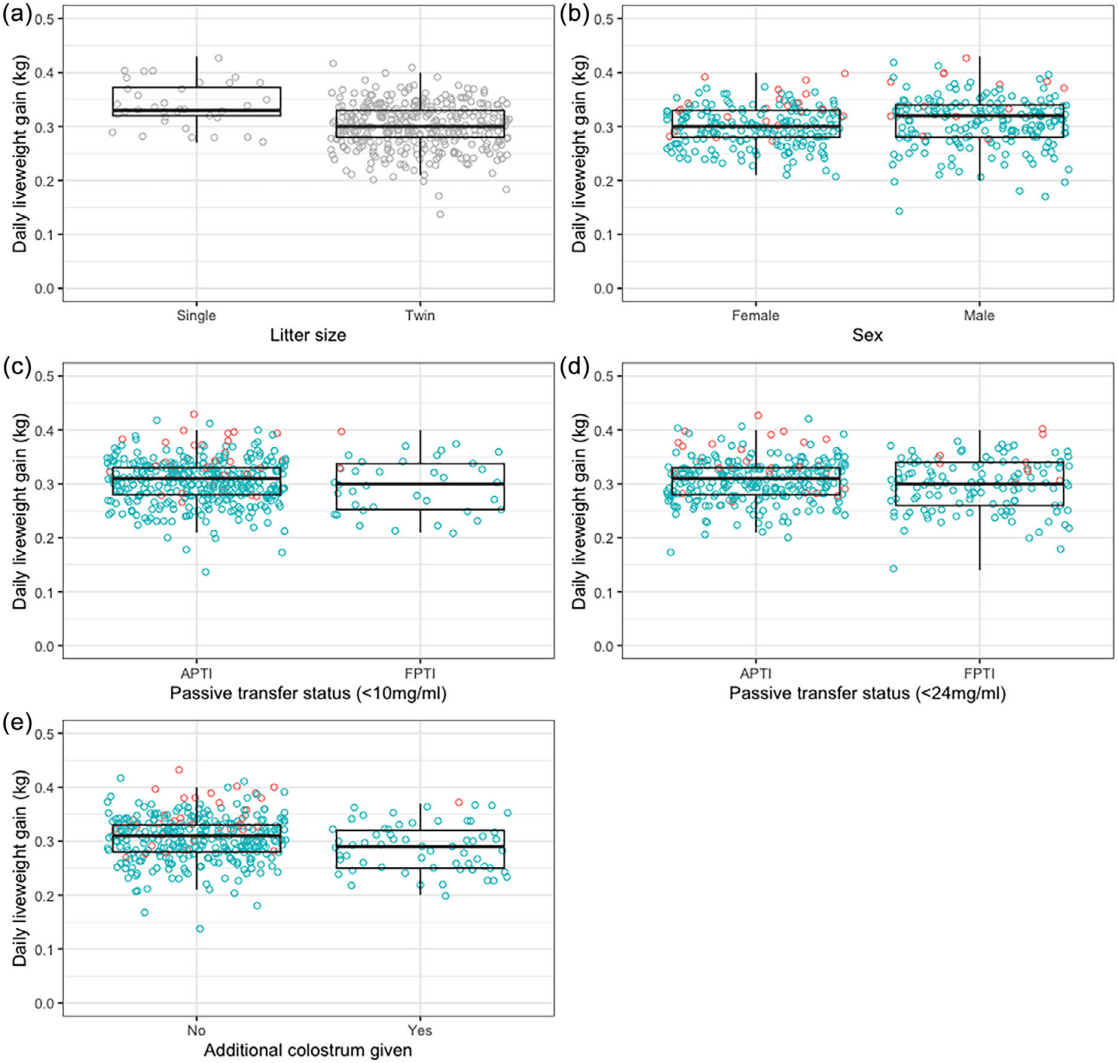
Lamb daily liveweight gain according to (a) reared litter size, (b) sex, (c) passive transfer status (≤10 mg/mL cut‐off), (d) passive transfer status (≤24 mg/mL cut‐off) and (e) whether additional colostrum had been given. Red dots: single lambs (*n* = 36); blue dots: twin lambs (*n* = 360). APTI: adequate passive transfer of immunity; FPTI: failure of passive transfer of immunity.

At weaning, male lambs were heavier than female lambs (*p* < 0.01), and singles were heavier than twins (*p* < 0.01) (Table 2). There was a weak relationship between birth weight and weaning weight (Figure 6; *r* ^2^ = 0.19). Lamb and ewe factors that may have influenced lamb DLWG were initially explored using univariable analysis with DLWG as a continuous variable. Singles had a higher DLWG than twins (*p* < 0.01). However, lamb sex, passive transfer status, provision of additional colostrum and oral antibiotic administration had no significant effect on DLWG (Figure 7; *n* = 396, *p* ≥ 0.05). Ewe factors (metabolic status, BCS, age and scan result) were not significantly associated with lamb DLWG. Relationships between factors did not alter when single lambs were removed from the dataset (*n* = 360).

**FIGURE 8:**
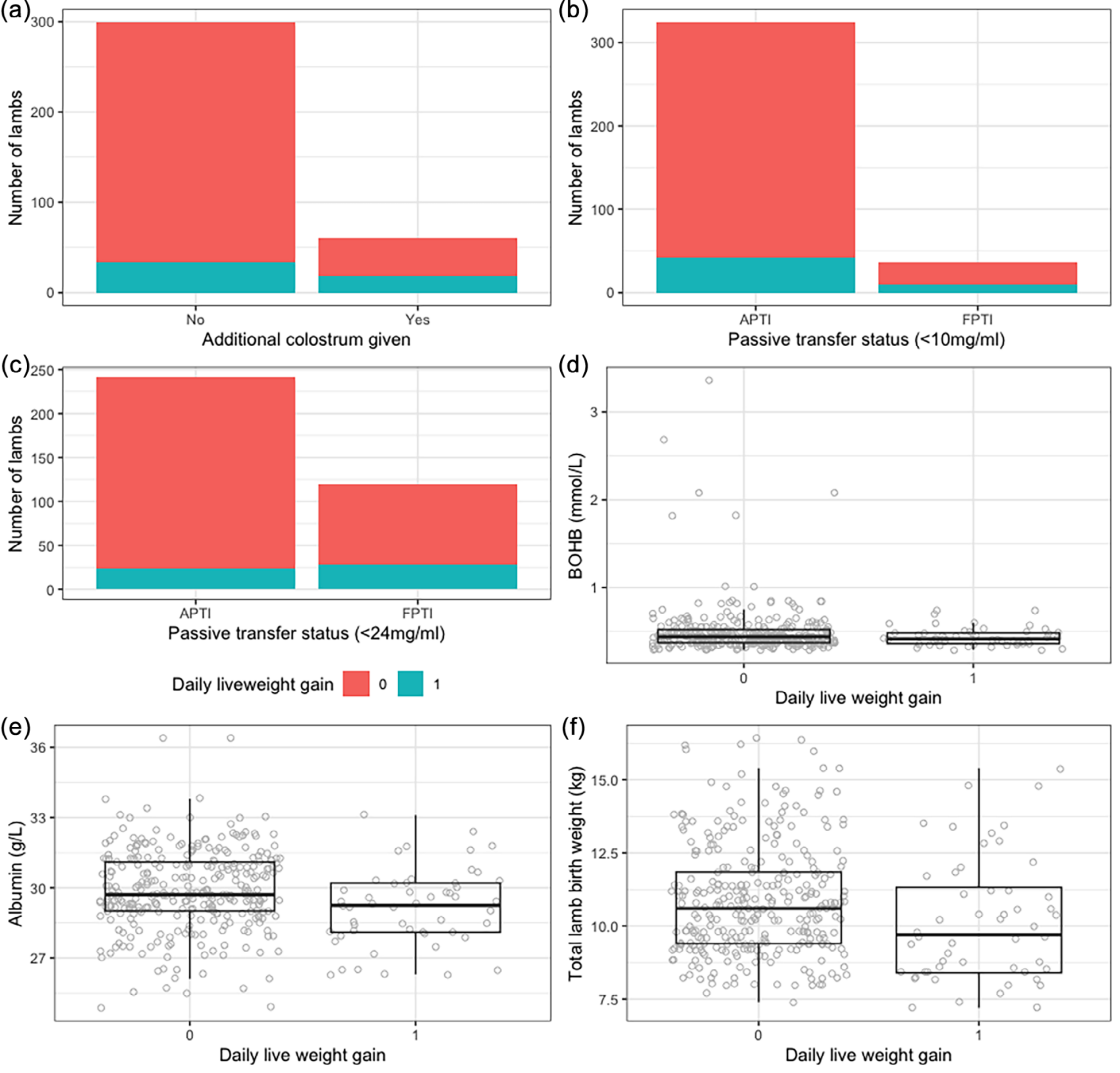
Factors with significant relationships with twin lambs having reduced daily liveweight gain (0: >0.26 kg/day; 1: <0.26 kg/day). (a) Whether additional colostrum had been given, (b) passive transfer status (≤10 mg/mL cut‐off), (c) passive transfer status (≤24 mg/mL cut‐off), (d) ewe blood beta‐hydroxybutyrate concentration pre‐lambing, (e) ewe blood albumin concentration pre‐lambing and (f) ewe total litter birth weight. APTI: adequate passive transfer of immunity; FPTI: failure of passive transfer of immunity.

To investigate whether it was possible to identify factors that were predictive of particularly poor lamb performance, DLWG was converted to a dichotomous variable. Reduced DLWG was defined as a DLWG of less than 0.26 kg/day, as this was the mean DLWG for the bottom third of lambs. Single‐reared lambs were excluded, as all lambs with reduced DLWG were twins (*n* = 360). Univariable analysis explored the relationship between reduced DLWG and the same lamb and ewe factors as for the analysis for DLWG as a continuous variable. Lambs provided with additional colostrum (*p* < 0.01), lambs with FPTI (IgG concentration < 24 mg/mL; *p* < 0.01), increased total litter weight at birth (*p* < 0.05), increased ewe blood BOHB concentration (*p* < 0.01) and low ewe blood albumin concentration (*p* < 0.01) prior to lambing were included in the maximal MLR model (Figure 8). For passive transfer status, 24 mg/mL or less was chosen as the IgG cut‐off due to it having a lower *p*‐value during univariable analysis than the IgG cut‐off of 10 mg/mL or less (*p* < 0.01 vs. <0.05). In the final MLR model, only additional colostrum supplementation and passive transfer status were retained as fixed effects and ewe as a random effect. Twin lambs with serum IgG concentrations of 24 mg/mL or less had increased odds (OR = 4.75, 1.17‒19.19, *p* < 0.05) of having reduced DLWG (Table 6). Notably, oral antibiotic administration and other lamb and ewe factors were not associated with reduced DLWG.

**TABLE 6:** Results of the final multivariable logistic regression model to identify variables associated with a daily liveweight gain of less than 0.26 kg/day in twin‐reared lambs (*n* = 360).

Variable	Level	OR (95% CI)	*p*‐value
Additional colostrum given	No	Base	
	Yes	3.79 (0.65‒21.94)	0.14
Passive transfer status (≤24 mg/mL)	Adequate	Base	
	Failure	4.75 (1.17‒19.19)	<0.05

Abbreviations: CI, confidence interval; OR, odds ratio.

## DISCUSSION

The flock used in this study is an example of a well‐managed UK lowland flock with favourable production metrics, and the results should be interpreted in this context. All losses between scanning and the immediate neonatal period (24 hours postpartum) in this flock were associated with ewes with multiple lambs (9.5% twins and 37% triplets). Due to the study design, we were unable to attribute these losses to the last trimester of pregnancy versus the immediate neonatal period. However, the dataset did allow exploration of the influence of ewe metabolic status on total lamb losses and FPTI during this period. The mean lamb DLWG of 0.30 kg is higher than the top third of lowland farms in the UK.^27^ Despite low lamb morbidity and mortality overall, serum IgG concentration at 24 hours of age or less and average DLWG until weaning varied significantly within the lamb cohort. With this in mind, we decided to focus on the poorest performing lambs in the flock, that is, those with a DLWG below 0.26 kg/day.

### Relationship between ewe nutritional status and lamb losses

In multiparous ewes, after accounting for litter size, increased blood albumin concentration was associated with decreased odds of losing a lamb between scanning and 24 hours postpartum. Blood albumin concentration in ewes is used as a measurement of long‐term protein status.^28^ Although limited longitudinal data have been published, blood albumin concentration generally decreases during pregnancy, particularly in the latter stages of pregnancy,^29^, ^30^ due to increased fetal growth and preparation for lactation.^31^ Furthermore, experimentally protein‐restricted ewes (60% of metabolisable protein requirements) have been shown to have smaller lambs.^32^ As approximately 70% of fetal growth occurs in the last trimester of pregnancy,^33^ inadequate supply of dietary protein may lead to depletion of maternal body protein and lamb losses. BCS is a measure of long‐term metabolic status and, combined with predicted litter size and metabolic profiling, can be used to guide feeding and management in the last trimester.^34^ Ewe BCS has been demonstrated to be associated with blood albumin concentration,^28^ and assessing ewe BCS prior to the last trimester is recommended to tailor nutritional requirements of different litter sizes.^7^, ^33^, ^35^ However, manual assessment of BCS is influenced by both fat and muscle cover and so cannot distinguish between ewes in adequate long‐term energy balance but negative long‐term protein balance and ewes in both adequate long‐term energy and protein balance. As such, other factors have been reported to also be associated with decreased blood albumin in ewes, including increased age,^36^ genotype^33^ and the presence of chronic diseases such as lameness or chronic fasciolosis.^37^, ^38^ With a range of factors affecting ewe blood albumin, it is not possible to establish a causal relationship between lower ewe blood albumin concentrations and the increased lamb losses identified in this study. That said, it is noteworthy that the majority of the ewes in this study were in target body condition, with blood BOHB levels that were largely normal. Future work should therefore investigate the relationships between lamb outcomes and ewe BCS, blood albumin concentration and disease status across different management systems in the UK.

### Relationship between ewe nutritional status and lamb FPTI

There are few studies that have investigated the variation in neonatal lamb serum IgG. The variation in lamb serum IgG at 8‒24 hours postpartum suggests that uptake of colostrum varied within litters and across the entire cohort. This variation is not necessarily surprising, as multiple ewe and lamb factors influence the uptake of colostrum.^39^ However, this variation offers the prospect of investigating factors that could be controlled through changes in husbandry practices to reduce FPTI. Using cattle serum IgG cut‐off values for FPTI, 10.4% and 33.2% of the lambs in the flock studied were defined as having ‘complete’ FPTI and ‘complete or partial’ FPTI, respectively (complete: ≤10 mg/mL serum IgG; partial: ≤24 mg/mL serum IgG). It is interesting to note that these proportions are comparable to the prevalence of FPTI reported in British suckler herds.^40^ Although further work is required to define a specific cut‐off value for lambs, it is likely that a significant proportion of these lambs received inadequate amounts of colostrum, which may be associated with poorer future health and production.

As lambs are born hypogammaglobulinaemic,^9^ passive transfer of immunity needs to be acquired 8‒24 hours after birth,^41^ and this is related to the speed, quality and quantity of colostrum consumed.^42^ Triplet lambs will be under increased competition to suckle from an ewe's udder, which may result in decreased colostrum uptake, leading to weaker lambs. Furthermore, previous evidence has indicated that lambs from larger litters have impaired behavioural development at birth, which may be associated with their ability to suckle.^43^ Smaller lambs or those in larger litters with increased competition to suckle may also lose heat more quickly and suffer from hypothermia, reducing their ability to suckle and resulting in reduced colostrum intakes.^44^ It is therefore not surprising that this study identified that triplet lambs had increased odds of having FPTI (≤24 mg/mL serum IgG). We also identified that lambs with serum IgG concentrations of 24 mg/mL or less were at increased odds of being born to an ewe that had an elevated BOHB concentration prior to lambing; hence, indicative of an association between short‐term energy balance prior to lambing and FPTI.^39^, ^43^ Various factors will affect the quantity and quality of colostrum produced by individual ewes. With lamb serum IgG as a measure of passive transfer of immunity from colostrum, this study identified a significant association with metabolic status prior to lambing. Colostrum yield is known to be dependent on adequate supplies of energy and protein in the last 3 weeks before lambing.^44^ Increased blood BOHB (i.e., ketonaemia) and the clinical presentation ‘pregnancy toxaemia’ are a consequence of negative energy balance due to an elevated energy requirement for fetal growth in the face of a suppression of feed intake.^45^ Clinical or subclinical ketonaemia may delay the onset of lactogenesis and reduce colostrum quantity and quality.^46^ As a consequence, lambs may have reduced colostrum intake and increased utilisation of brown fat reserves, resulting in weak hypothermic lambs. Ewes with subclinical pregnancy toxaemia are also more likely to develop mastitis,^47^ which could lead to ewes being unable to nurse their lambs and further hinder colostrum intakes. An association with FPTI was not seen when using serum IgG of 10 mg/mL or less as the cut‐off, which is likely due to most ewes in this study having adequate energy status (Figure 2) rather than the absence of this biological relationship in this flock. Interestingly, while ewe blood albumin concentration was associated with lamb losses in this study, it was not associated with lamb passive transfer status, suggesting that the mechanism responsible for this association is independent of colostrogenesis by the ewe and immunoglobulin uptake by the lamb.

### Relationship between FPTI and lamb growth

Compared to studies in calves,^48^ there have been few studies that have attempted to define serum IgG cut‐off values for FPTI in lambs, which could be used to investigate the association between passive transfer status and lamb outcomes. One study has identified a weak negative association (*r* ^2^ = 0.18) between serum immunoglobulin concentration and faecal staining in a cohort of lambs at 48‒72 hours after birth,^49^ highlighting the relationship between passive transfer status and enteric disease. A separate study of dairy lambs has demonstrated a weak positive relationship (*r* ^2^ = 0.26) between serum IgG concentrations and mean DLWG until 28 days of age.^50^ However, neither study proposed an IgG concentration cut‐off to define FPTI in lambs. As such, our study sought to identify a relationship between FPTI and lamb outcomes (morbidity, mortality and DLWG until weaning) using thresholds validated in beef calves. Beef calves with serum IgG concentrations of 24 mg/mL or less are 1.6 times more likely to become ill before weaning and 2.7 times more likely to die before weaning.^51^ In our study, lamb morbidity (2.6%) and mortality (4.1%) between 24 hours of age and weaning were too low to warrant further analysis in a flock of this size. This highlights the difficulties of conducting such studies in the UK, where blood sampling on this scale is not considered to represent recognised animal husbandry or recognised veterinary practice.

Using the serum IgG cut‐off of 24 mg/mL or less to define FPTI, we identified that twin lambs with FPTI had increased odds of having a lower DLWG (<0.26 kg/day). Although less than 0.26 kg/day may be acceptable in some systems, this highlights a potential loss of revenue in this high‐performing lowland flock, with increased rearing and feeding costs for these lambs. Other lamb/ewe and neonatal management factors were not associated with lower DLWG; however, it is important to note that most ewes in this study were in good body condition and their metabolic status was generally good. Consequently, these results do not contradict the significant body of work that exists showing that poor ewe body condition and metabolic status are associated with poorer lamb outcomes. Among the lamb/ewe and neonatal factors investigated, increased total litter weight at birth, increased ewe pre‐lambing plasma BOHB concentration and low ewe pre‐lambing plasma albumin concentration ceased to be significant during multivariable modelling and therefore were removed during model selection. Specifically, neither litter size at birth nor total litter birthweight was predictive of DLWG. As lambs born as triplets were reared as twins, with the third lamb artificially reared, this would suggest that any association of litter size at birth on DLWG is exerted via competition for maternal resources during rearing rather than conditions in utero. The fact that prepartum ewe plasma BOHB and albumin concentrations were not retained in the final MLR model may suggest that these ewe factors exert their effect on DLWG indirectly via their influence on lamb passive transfer status. Despite not being statistically significant, colostrum supplementation was retained in the final MLR model. This may indicate that stockpersons are relatively good at identifying lambs that go on to be at increased risk of poorer DLWG. However, despite supplementation with additional colostrum, these lambs still go on to be at increased risk of poorer DLWG. This is analogous to work in calves, showing that supplementation with colostrum is a risk factor for FPTI;^40^ that is, stockpersons are good at identifying calves at risk of FPTI, but the intervention is insufficient to achieve adequate passive transfer status. Overall, using the beef calf serum IgG cut‐off of 24 mg/mL or less to define FPTI highlights its potential use in exploring the relationship between FPTI and lamb outcomes in field studies in a range of management systems, including the influence of variation in colostrum quality, quantity and timing of consumption.

Despite the perceived risk of watery mouth on this farm, it was notable that there was no association between preventive oral antibiotic therapy at birth and lamb outcomes. It is worth highlighting that routine oral antibiotic treatment was not a standard practice in this flock; therefore, lambs were not denied an existing management practice. Furthermore, the randomisation of antibiotic treatment in this flock was conducted under a Home Office licence, highlighting the challenges of undertaking such studies in the UK. We cannot exclude the low levels of morbidity and mortality in this flock being due to the administration of preventive oral antibiotics to half of the lambs born; however, we did demonstrate that its use had no effect on lamb growth rates, and that lambs treated with a placebo did not suffer from significantly higher levels of morbidity and mortality than antibiotic‐treated lambs. The UK sheep industry has generally low antibiotic use compared to other livestock systems; however, this study provides evidence for where usage could be reduced further, especially considering the removal of licensed oral antibiotic products for neonatal lambs from the UK market since this study was conducted.^52^

## CONCLUSION

This study's findings highlight the need to focus on ewe metabolic status in late pregnancy and colostrum management within the first 8‒24 hours of life. Ewes bearing multiple fetuses had an increased risk of losing a lamb, and the more lambs born in a litter, the more likely they were to suffer from FPTI. Once the effect of litter size on ewe metabolic status had been controlled for, ewe metabolic parameters were also associated with these outcomes. We also identified an association between FPTI and DLWG, although further work is required to define a consensus cut‐off(s) for FPTI across lamb production systems to inform this evidence‐based approach for benchmarking on individual farms. This study also provides further evidence that blanket prophylactic neonatal oral antibiotic administration is not necessary in a well‐managed flock.^51^

In conclusion, veterinarians are well placed to develop approaches to reduce the incidence of FPTI through proactive flock health planning. However, there is a need to better articulate the association of FPTI at the individual farm level through improved data collection to inform targeted evidence‐based control measures.^53^

## AUTHOR CONTRIBUTIONS

Alexander Corbishley, Rob F. Kelly, Amy Jennings, Fiona M. Lovatt, Peers L. Davies, Elizabeth Burrough, Jennifer S. Duncan, Robert M. Hyde, Andy Hopker, Katie Adam and Ann Bruce conceived the original project. Alexander Corbishley and Rob F. Kelly designed the field study, sample design and databases associated. Rob F. Kelly, Alexander Corbishley and Amy Jennings developed the field SOPs and collected the data. Rob F. Kelly, Alexander Corbishley and Elizabeth Burrough conducted laboratory work. Rob F. Kelly, Alexander Corbishley and Geraldine Russell cleaned the initial dataset. Rob F. Kelly, Alexander Corbishley and Amy Jennings contributed to the analysis. Rob F. Kelly was responsible for writing the initial drafts. Rob F. Kelly, Amy Jennings, Emily Gascoigne, Peers L. Davies, Jennifer S. Duncan, Andy Hopker, Robert M. Hyde, Fiona M. Lovatt, Ann Bruce and Alexander Corbishley contributed to the final draft.

## CONFLICT OF INTEREST STATEMENT

The authors and participants in this study have no other conflicts of interest.

## ETHICS STATEMENT

The work was reviewed and approved by the Animal Welfare & Ethical Review Body at R(D)SVS and conducted under licence in accordance with the Animals Scientific Procedures Act 1986.

## Data Availability

The data that support the findings of this study are available from the corresponding author upon reasonable request.
